# Supplementary material for: An Assessment of Human Opportunistic Pathogenic Bacteria on Daily Necessities in Nanjing City during Plum Rain Season
Source: Microorganisms. 2024 Jan 26;12(2):260. doi: 10.3390/microorganisms12020260 (PMC10892523; doi:10.3390/microorganisms12020260)
Supplement: Supplementary file 1 [file microorganisms-12-00260-s001.zip › microorganisms-2793788-supplementary.pdf]

Supplementary materials

An Assessment of Human Opportunistic Pathogenic Bacteria on Daily Necessities in Nanjing City during Plum Rain Season

Xiaowei Yu, Yifan Yin, Zuoyou Wu and Hui Cao

Table S1 The specific situation of Nanjing's plum rain season in the past ten years.

Year	Time point into the plum rain	Time point out of the plum rain	Length of the plum rain season/d	Precipitation in the plum rain season/mm	Annual precipita tion/mm	Proportion of precipitation in the plum rain season to the annual precipitation/%
2013	23 June	8 July	16	255.0	898.4	28.4
2014	26 June	18 July	24	217.0	1091.1	19.9
2015	24 June	13 July	20	374.7	1765.6	21.2
2016	19 June	20 July	32	597.3	1807.7	33.0
2017	22 June	11 July	20	134.8	1126.3	12.0
2018	22 June	9 July	18	173.2	1088.1	15.9
2019	18 June	21 July	33	127.3	578.4	22.0
2020	10 June	2 August	53	659.0	1260.2	52.3
2021	18 June	10 July	22	260.4	1042.0	25.0
2022	23 June	11 July	18	180.9	1106.5	16.3

Table S2 Characteristics of the plum rain season and seasons in 2022.

Season	Average temperature/℃	Average relative humidity/%	Precipitation/mm
Spring	17.7	62.0	81.0
Summer	29.8	66.3	163.3
Autumn	18.3	68.3	68.7
Winter	13.4	66.0	45.0
plum rain season	29.4	71.0	181.0

March, April and May are considered spring, June, July and August are considered summer, September, October and November are considered autumn, and December, January and February are considered winter.

Table S3 Proportion of biological correlates of human opportunistic pathogenic bacteria.

Species	C-NPR/%	C-PR/%	E-NPR/%	E-PR/%	R-NPR/%	R-PR/%	W-NPR/%	W-PR/%
Stenotrophomonas maltophilia	41.21	44.35	31.89	24.28	23.09	37.51	40.56	51.59
Staphylococcus aureus	11.33	10.06	15.18	17.58	37.78	32.58	13.26	12.21
Pseudomonas aeruginosa	11.83	11.42	15.46	16.21	6.86	8.3	10.7	10.21
Proteus mirabilis	0	0.19	NA	1.11	1.22	0.17	NA	NA
Mycobacterium tuberculosis	2.22	2.01	16.32	11.9	0.19	1.9	8.79	6.47
Listeria monocytogenes	3.03	2.33	0.49	0.56	1.35	2.07	0.26	1.14
Legionella pneumophila	1.65	5.21	NA	NA	5.39	NA	NA	NA
Klebsiella pneumoniae	19.29	16.98	14.1	6.96	6.16	8.04	18.83	12.41
Haemophilus influenzae	NA	0.71	3.42	4.58	NA	NA	0.05	0.85
Enterococcus faecium	6.37	4.66	3.04	10.72	17.58	5.7	5.57	1.87
Bacillus cereus	3.06	2.07	0.11	6.11	0.38	3.72	1.99	3.25

The "PR" represents "plum rain", "NPR" represents "non-plum rain" and "NA" means that this human opportunistic pathogenic bacterium had no biological correlations.

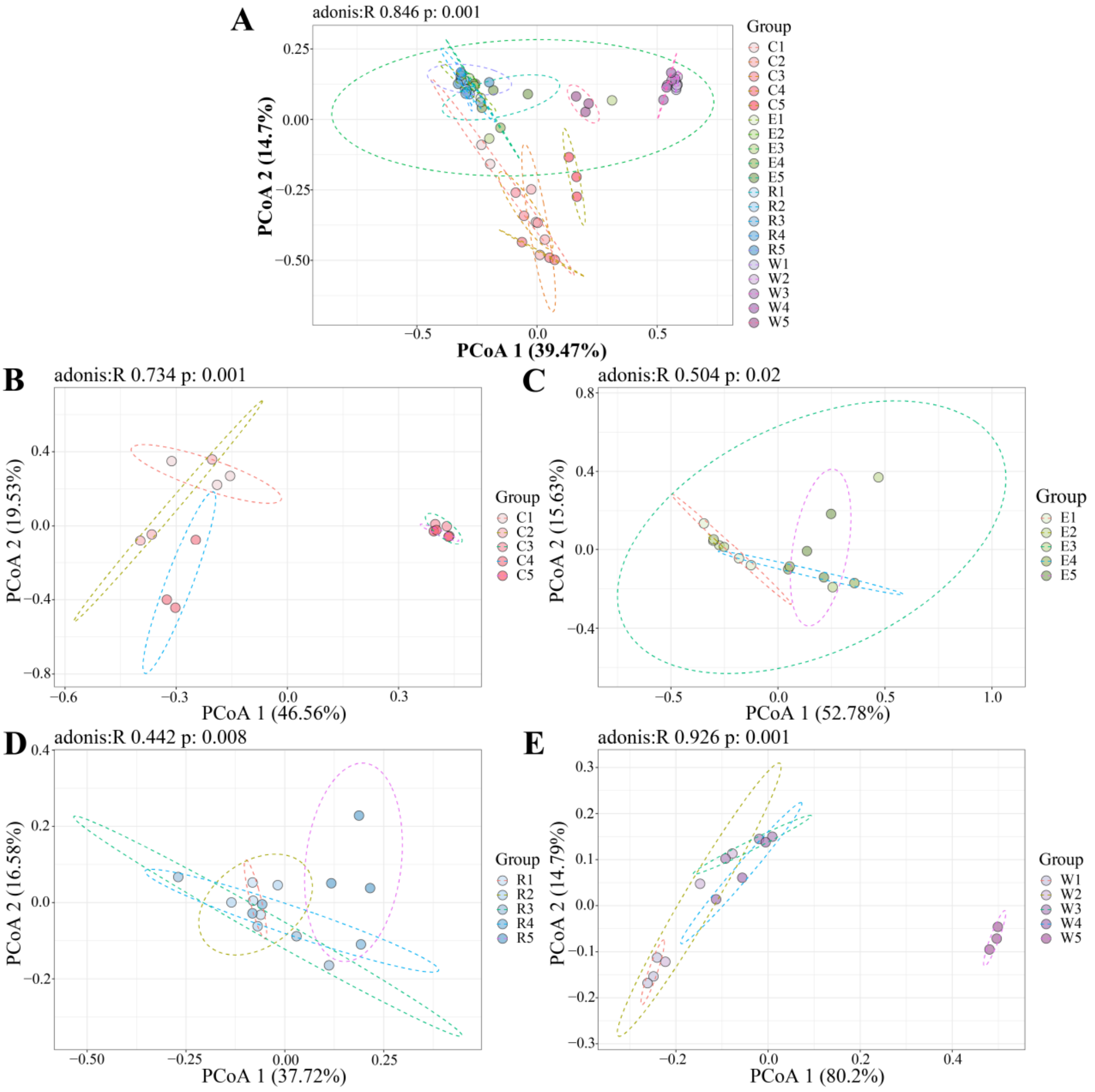

Figure S1. Principal coordinate analysis (PCoA) of human opportunistic pathogenic bacterial community based on Bray-Curtis distance metrics. (A) All daily necessities; (B) cotton cloth; (C) electric bicycles; (D) rice and (E) washbasins.

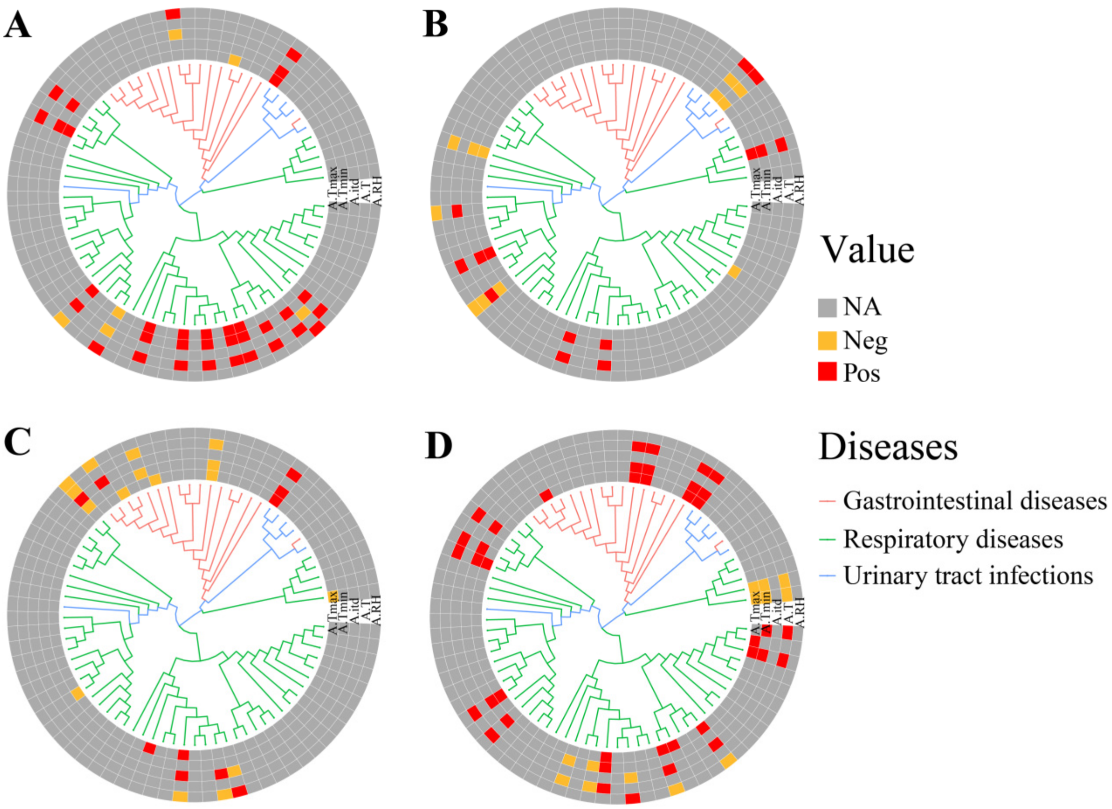

Figure S2. Phylogenetic tree heatmap of human opportunistic pathogenic bacteria associated with environmental factors on daily necessities: **(A)** cotton cloth, **(B)** electric bicycles, **(C)** rice and **(D)** washbasins. For abbreviations see Table 1.
